# Endometrial carcinoma risk among women diagnosed with endometrial hyperplasia: the 34-year experience in a large health plan

**DOI:** 10.1038/sj.bjc.6604102

**Published:** 2007-11-20

**Authors:** J V Lacey, O B Ioffe, B M Ronnett, B B Rush, D A Richesson, N Chatterjee, B Langholz, A G Glass, M E Sherman

**Affiliations:** 1 Hormonal and Reproductive Epidemiology Branch, Division of Cancer Epidemiology and Genetics, National Cancer Institute, Rockville, MD, USA; 2 Department of Pathology, University of Maryland Medical Center, Baltimore, MD, USA; 3 Department of Pathology, The Johns Hopkins Medical Institutions, Baltimore, MD, USA; 4 Kaiser Permanente Center for Health Research, Portland, OR, USA; 5 Biostatistics Branch, Division of Cancer Epidemiology and Genetics, National Cancer Institute, Rockville, MD, USA; 6 Division of Biostatistics, Department of Preventive Medicine, Keck School of Medicine of the University of Southern California, Los Angeles, CA, USA

**Keywords:** endometrial adenocarcinoma, uterine cancer, atypical hyperplasia, counter-matching, histopathology

## Abstract

Classifying endometrial hyperplasia (EH) according to the severity of glandular crowding (simple hyperplasia (SH) *vs* complex hyperplasia (CH)) and nuclear atypia (simple atypical hyperplasia (SAH) *vs* complex atypical hyperplasia (CAH)) should predict subsequent endometrial carcinoma risk, but data on progression are lacking. Our nested case–control study of EH progression included 138 cases, who were diagnosed with EH and then with carcinoma (1970–2003) at least 1 year (median, 6.5 years) later, and 241 controls, who were individually matched on age, date, and follow-up duration and counter-matched on EH classification. After centralised pathology panel and medical record review, we generated rate ratios (RRs) and 95% confidence intervals (CIs), adjusted for treatment and repeat biopsies. With disordered proliferative endometrium (DPEM) as the referent, AH significantly increased carcinoma risk (RR=14, 95% CI, 5–38). Risk was highest 1–5 years after AH (RR=48, 95% CI, 8–294), but remained elevated 5 or more years after AH (RR=3.5, 95% CI, 1.0–9.6). Progression risks for SH (RR=2.0, 95% CI, 0.9–4.5) and CH (RR=2.8, 95% CI, 1.0–7.9) were substantially lower and only slightly higher than the progression risk for DPEM. The higher progression risks for AH could foster management guidelines based on markedly different progression risks for atypical *vs* non-atypical EH.

Abnormal vaginal bleeding often prompts endometrial biopsy or curettage to exclude endometrial carcinoma and endometrial hyperplasia (EH), its precursor (Clark *et al*, 2002; Montgomery *et al*, 2004). Endometrial hyperplasia is a pathologically heterogeneous diagnosis that ranges from histologically subtle and spontaneously reversible proliferative lesions to incipient carcinoma (Montgomery *et al*, 2004; Mazur, 2005). To describe the risk of subsequent carcinoma (Marsden and Hacker, 2001; McCluggage, 2006), the widely used World Health Organization (WHO) system classifies biopsy diagnoses of EH according to the severity of glandular crowding and the presence of nuclear atypia (Clark *et al*, 2006). Lesions showing minimal crowding are considered simple hyperplasia (SH), whereas lesions showing greater crowding are designated as complex hyperplasia (CH) (Zaino, 2000). Simple hyperplasia and CH with nuclear atypia are designated as simple atypical hyperplasia (SAH) and complex atypical hyperplasia (CAH), respectively. Complex atypical hyperplasia is more common than SAH and represents the most frequently identified immediate precursor of endometrial carcinoma (Silverberg, 2000).

Distinguishing low-risk EH lesions that can be conservatively managed with progestogen-based treatment plus surveillance from higher-risk EH lesions that require immediate surgical treatment has clinical implications (Clark *et al*, 2006; McCluggage, 2006; Soslow, 2006). There is no consensus on the management of SH or CH (Clark *et al*, 2006), which make up most EH diagnoses, but these lesions are thought to pose only modest risk of progression to carcinoma (Marsden and Hacker, 2001; Montgomery *et al*, 2004). In contrast, hysterectomy is generally recommended for women with SAH or CAH because of a high probability of underlying carcinoma when AH is diagnosed (ACOG, 2005). Approximately 20 200 women in the United States who undergo hysterectomy receive a primary hospital discharge diagnosis of EH each year (Keshavarz *et al*, 2002), but progression risks for EH have not been accurately characterised in rigorous population-based studies (Silverberg, 2000). Current management of EH relies on largely historical data from studies that lacked adequate control groups (Kurman *et al*, 1985; Feldman *et al*, 1995; Terakawa *et al*, 1997; Tabata *et al*, 2001; Horn *et al*, 2004; Baak *et al*, 2005) and were limited by sample size (Feldman *et al*, 1995; Tabata *et al*, 2001), short follow-up (Feldman *et al*, 1995; Terakawa *et al*, 1997; Tabata *et al*, 2001), suboptimal statistical methods (Kurman *et al*, 1985; Pettersson *et al*, 1985; Feldman *et al*, 1995; Terakawa *et al*, 1997; Tabata *et al*, 2001; Horn *et al*, 2004), and minimal clinical and treatment information (Kurman *et al*, 1985; Pettersson *et al*, 1985; Terakawa *et al*, 1997; Tabata *et al*, 2001; Horn *et al*, 2004). Endometrial hyperplasia diagnoses can misclassify disease severity because of biopsy sampling errors (Zaino, 2000; Trimble *et al*, 2006; Zaino *et al*, 2006) or the community pathologists’ reported tendency to overestimate lesion severity (Silverberg, 2000). Accurate and precise estimates of endometrial carcinoma risk after an EH diagnosis are needed to develop evidence-based management guidelines. Therefore, we conducted a nested case–control study of EH progression risk.

## MATERIALS AND METHODS

### Study setting

The Kaiser Permanente Center for Health Research (KPCHR) is the research arm of the Kaiser Permanente Northwest (KPNW) pre-paid health plan (Wagner *et al*, 2005). Since the early 1940s, the KPNW has provided essentially all health care to its members, who are drawn from the Portland OR metropolitan area. We described previously our study design and methods in detail (Lacey, submitted).

Unique KPNW identification numbers allow linkage across computerised administrative, surgical, pharmacy, tissue archive, and tumour registry databases. The tumour registry captures 95–98% of all newly diagnosed cancers among KPNW members. The KPNW Department of Pathology annually diagnoses approximately 50 endometrial carcinomas and interprets 1100 endometrial biopsies, 8% of which are reported as EH.

### Cases

Using these databases, we identified 188 potential cases: women who received a diagnosis of incident EH on biopsy or curettage, followed by a diagnosis of incident endometrial carcinoma at least 1 year later, between 1 August 1970 and 31 December 2003. To capture as many potential progression cases as possible, we also included 41 women with an index biopsy diagnosed as non-atypical EH (SH or CH) who received a diagnosis of CAH at hysterectomy at least 1 year later. We henceforth refer to biopsy or curettage as biopsy (Shutter and Wright, 2005) and to the initial diagnosis of EH as the index biopsy.

### Slide review

We retrieved all pathology slides and reports for all index biopsies, follow-up biopsies, and hysterectomy procedures. One pathologist (MES) initially reviewed all slides, assigned a WHO classification, and selected one representative slide for each specimen. Two gynaecologic pathologists (BMR and OBI), who were masked to all specimen data except patient age and accession date, then independently classified the selected slides according to agreed-upon WHO nomenclature (Kurman *et al*, 1985). We assigned a panel diagnosis for each specimen based on exact agreement between at least two of the three pathologists. When all three reviewers disagreed, the first review diagnosis (MES's) became the panel diagnosis. Panel diagnoses were inactive endometrium; atrophy; polyp; secretory endometrium (SEM); proliferative endometrium (PEM); disordered proliferative endometrium (DPEM); SH; CH; AH (SAH or CAH); EH, not otherwise specified (NOS); or carcinoma. For analysis, we combined inactive, atrophy, polyp, SEM, and PEM into ‘negative’ and considered DPEM to represent equivocal EH (Silverberg, 2000; Mazur, 2005; McCluggage, 2006).

We excluded eight potential cases with miscoded original diagnoses of EH (*N*=6) or cancer (*N*=2) and seven cases whose panel diagnosis did not confirm their original end point diagnosis of CAH or carcinoma at hysterectomy. Of the remaining 214 potential cases, 138 (65.4%) with index biopsy panel diagnoses of DPEM (*N*=33), SH (*N*=42), CH (*N*=21), or AH (*N*=42) were eligible. This included 28 cases whose original index biopsy was downgraded by the panel but who had a subsequent biopsy that met all eligibility criteria. The 76 ineligible cases had index biopsies with panel diagnoses of inactive (*N*=14), atrophy (*N*=8), polyp (*N*=4), SEM (*N*=8), PEM (*N*=22), or carcinoma (*N*=13); or their slides were unsatisfactory (*N*=7) or unavailable (*N*=1) for review. Of the eligible cases, 127 clinically progressed from EH to carcinoma and 11 clinically progressed to AH at hysterectomy (Supplementary Table 1).

### Controls

For each case, we constructed a risk set of individually matched potential controls: all KPNW members with an index biopsy diagnosis of incident EH who remained at risk (i.e. no hysterectomy or uterine carcinoma) for at least as long as the progression interval of the case to whom they were matched. Each control within each risk set was matched to that risk set's case on age at (±1 year) and date of (±1 year) index biopsy and assigned a matched censoring date based on the case's progression interval. When necessary, we relaxed the date- and age-matching criteria in successive 1-year intervals, up to 5 years, to populate risk sets that were initially empty. To avoid selecting controls who were diagnosed with endometrial carcinoma shortly after their matched censoring date, we required each potential control to remain at risk for one additional year after her matched censoring date. Therefore, the controls who were eligible to be selected had all been diagnosed with EH at the same age and date as the cases and had the same duration of at-risk follow-up as the cases.

Almost three-quarters of all EH diagnoses at the KPNW are SH (Lacey JV Jr, unpublished observation). Random selection of controls would have led to underrepresentation of patients who had CH or AH, especially in risk sets for older cases or cases with longer progression intervals. To avoid this potential limitation, we selected controls via counter-matching, a form of weighted random sampling (Lacey, submitted). Counter-matching (Langholz and Clayton, 1994) oversamples controls with rare values of a proxy exposure that is known for the entire cohort. Detailed exposures are then determined for the selected controls, rather than the entire cohort. The correlated proxy and detailed exposures increase statistical power while reducing data collection burdens (Cologne *et al*, 2004). Our proxy exposure was the original KPNW index biopsies diagnoses. We selected cases and controls based on those EH diagnoses (and thus all 229 potential cases could serve as controls for other eligible cases). Our detailed exposure was the pathology panel review diagnosis, which was used in our statistical analysis.

The 138 risk sets (one per eligible case) had 5891 total potential controls (mean=42.7 controls per risk set; range=4–123) who represented 2946 unique patients because controls could appear in multiple risk sets. To counter-match, we needed to know the original EH classifications for all 5891 potential controls. The KPNW pathology database recorded EH diagnoses, but EH classifications – SH, CH, or AH – were only available in the pathology reports. Reviewing 5891 pathology reports was impractical, so we used batch-quota sampling (Langholz, 2006) to review batches of pathology reports from each risk set to determine frequencies of SH, CH, and AH. We translated EH terminology used before 1995 (Silverberg, 2000) into WHO nomenclature as needed (e.g., ‘mild’ to SH, ‘moderate’ or ‘adenomatous’ to CH, and ‘severe’ to AH). We reviewed 3182 (54%) potential controls’ pathology reports: 2230 were SH (70%; includes 26 DPEM and 54 EH, NOS), 666 were CH (21%), and 153 were AH (5%; includes 10 SAH and 46 AH, NOS). We excluded 133 (4.2%) potential controls whose reports were miscoded as EH.

We selected three counter-matched controls from each eligible case's risk set. First, we chose two controls whose index biopsy original EH classification differed from the case's index biopsy original EH classification (e.g., if the case had CH, we chose one control who had SH and one control who had AH). Then, regardless of the case's original EH classification, we chose a third control who had AH. We intentionally oversampled controls with AH because we expected our pathology panel to downgrade many of those original AH diagnoses. If a risk set did not contain the desired AH control(s), we substituted a control originally classified as CH for the desired AH control. We selected 413 potential controls (one case had only two controls): 129 SH (31%), 153 CH (37%), and 131 AH (32%).

### Control slide review

**Figure 1:**
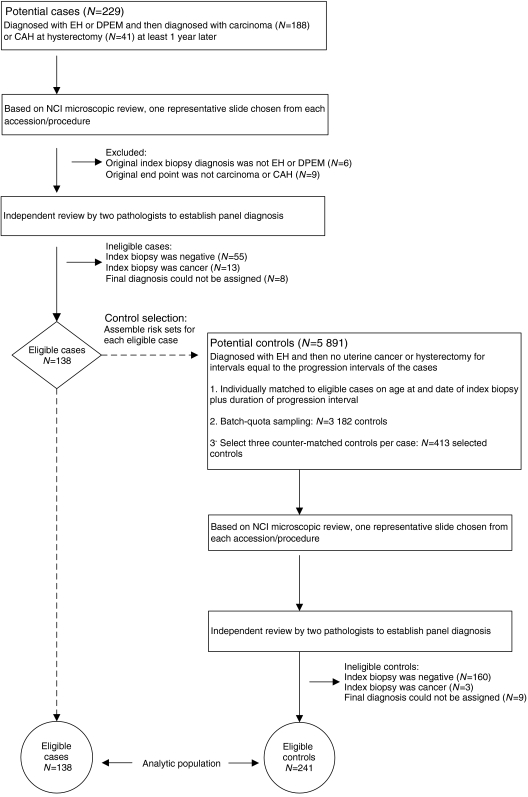
Identification, assessment, and eligibility status of potential cases and potential controls.

Our pathology panel review of controls’ slides employed the same protocol that was used for cases. Nine (2%) index biopsy slides were unavailable or unsatisfactory. Of the 404 (98%) controls whose slides were reviewed, 160 (39%) were ineligible because their index biopsies had final pathology panel diagnoses of negative (inactive, *N*=44; atrophic, *N*=7; polyp, *N*=2; SEM, *N*=24; PEM, *N*=83) or carcinoma (*N*=3). The 241 controls (58%) with index biopsy final diagnoses of DPEM (*N*=97; 24%), SH (*N*=67; 16%), CH (*N*=43; 10%), or AH (*N*=34; 8%) were eligible (Figure 1).

### Medical record review

We used a standardised abstract form to extract demographic characteristics, height and weight, reproductive and pregnancy history, other health factors, use of exogenous hormones, and treatment for EH. Risk factors were generally assessed at the time of index biopsy. We supplemented medical record data with computerised linkage to outpatient pharmacy data available after 1986. By 1993, 93–97% of KPNW members had pharmacy benefits at KPNW-operated pharmacies.

### Statistical analysis

Conditional logistic regression generated rate ratios (RRs) and 95% confidence intervals (CIs) to estimate the relative risk of being diagnosed with carcinoma after a diagnosis of EH. Risk estimates were based on the panel diagnoses of EH type, with DPEM as the reference group.

To assess confounding, we examined associations between EH type at index biopsy (Table 1) and endometrial carcinoma risk factors from the medical record data (see Table 2 for variables and categories). Only body mass index (BMI; <25, 25–34, ⩾35 kg m^−2^) and repeat biopsies (any within first 6 months, any subsequent follow-up biopsy, or none) were statistically significantly associated with both EH type and case–control status. Because of its clinical importance, we also adjusted for treatment (progestogen-based, other types, or none), even though treatment was similar for cases and controls (Table 1). Final regression models included sampling weights for both the batch-quota and counter-matched sampling (which were included as an offset in standard conditional logistic regression; Langholz, 2007) and were adjusted for age (in 1-year intervals), date (in 1-year intervals), duration of follow-up (in days), repeat biopsies, BMI, and treatment. Negative confounding by BMI and positive confounding by repeat biopsies essentially balanced out each other, while adjustment for treatment had minimal influence on the risk estimates.

### Human subjects

The KPCHR's Research Subjects Protection Office and the National Cancer Institute's Special Studies IRB approved this study.

### Role of the funding source

The funding source did not play a role in the study design; collection, analysis, or interpretation of data; manuscript writing; or decision to submit the manuscript for publication.

## RESULTS

Of the 127 cases diagnosed with endometrial carcinoma, 121 (95%) were endometrioid adenocarcinomas, five were clear cell carcinomas (4%), and one was a mucinous carcinoma (1%). Eighty-two percent were well differentiated and 11% had spread beyond the uterus.

Age, date, and length of progression interval were similar among eligible cases and controls, as expected from our matched design (Table 1). The overall median age at the time of index biopsy was 52 years. Median ages at the time of index biopsy were similar between cases and controls for each EH classification (data not shown).

The median interval between index biopsy and diagnosis of carcinoma was 6.7 years (range, 1–24.5). Cases’ intervals were longer for DPEM (10.4 years) and SH (8.6 years) than for CH and AH. For each EH classification, some cases had progression intervals longer than 10 years.

Three-fourths of cases and a slightly higher percentage of controls had at least one follow-up biopsy after the index biopsy but before the biopsy that prompted hysterectomy. Controls were twice as likely as cases to have had follow-up biopsies within 6 months of the index biopsy. However, total mean (2.2) and median (2) numbers of biopsies were similar. Over 80% of both groups received progestogen-based treatment during follow-up, including similar proportions of oral and injectable progestogens.

Age at menarche, parity, menopausal status, age at menopause, and smoking were similar between cases and controls (Table 2). Cases were significantly more likely than controls to have higher BMI and a history of irregular menses or diabetes before their index biopsy. Morbid obesity (BMI⩾40 kg m^−2^) was present in 21% of cases and 15% of controls. Oral contraceptive use and menopausal hormone therapy use were less common in cases than in controls. Approximately 60% of both cases and controls were pre- or perimenopausal at the time of their index biopsy.

Women diagnosed with EH were significantly more likely (RR=4.0, 95% CI, 2.0–7.7) than women diagnosed with DPEM to be subsequently diagnosed with carcinoma (Table 3). Increasing severity of EH was associated with a nearly exponential increase in progression risk. The RRs for SH and CH were 2.0 (95% CI, 0.92–4.5) and 2.8 (95% CI, 1.0–7.9), respectively, whereas the risk of carcinoma was over 10 times higher after an index biopsy of AH (RR=14.2, 95% CI, 5.3–38.0).

To improve the statistical precision of risk estimates by time interval since index biopsy, we expanded the reference group to include DPEM or SH (Table 4). Progression risks for AH were higher within 5 years of index biopsy (RR=48, 95% CI, 8–294) than after 5 or more years of follow-up (RR=3.5, 95% CI, 1.3–9.6); the CIs for these risk estimates barely overlapped. A diagnosis of CH did not significantly increase risk of carcinoma within or after 5 years.

### Other analyses

Results did not change after also adjusting for the number of progestogen prescriptions; restricting analyses to women with documented pharmacologic treatment or women who were postmenopausal or over 50 years of age at the time of index biopsy; or excluding the eight cases and one control who used tamoxifen before or during follow-up or the 11 cases (and their 18 matched controls) whose clinical end point was AH at hysterectomy.

Analyses that successively excluded risk sets with short progression intervals revealed the same pattern of risks. The RRs for SH, CH, and AH were 1.9, 2.8, and 14.6, respectively, after excluding progression intervals less than 2 years; 1.8, 1.9, and 9.8, respectively, after excluding progression intervals less than 3 years; and 1.6, 1.8, and 6.6, respectively, after excluding progression intervals less than 4 years. None of these RRs for SH or CH were statistically significant, whereas all of these RRs for AH were statistically significant (data not shown).

Progression risks were somewhat higher among women who had one or more follow-up biopsies. Many cases and controls had repeat biopsies within 3 or 6 months of their index biopsy; when risk was based on the most severe panel diagnosis for any biopsy within the first 3 or 6 months of follow-up, results did not materially change (data not shown).

## DISCUSSION

Rigorous estimates of progression risks for EH could foster improved clinical management of abnormal vaginal bleeding. Numerous methodologic deficiencies limit the available data on progression. In our analysis, biopsy diagnoses of SH and CH did not substantially increase the risk of progression to carcinoma compared with DPEM. Thus, DPEM, SH, and CH seem to be low-risk lesions that may be amenable to attempts at conservative management with close surveillance. Progression risks for AH were substantially elevated and remained increased for years. Our findings support the development of a simplified, potentially dichotomous, pathologic classification that would differentiate non-atypical EH from AH.

Risk of carcinoma after AH was markedly higher 1–5 years after index biopsy and remained significantly elevated well after 5 years. Most of the women who received a panel diagnosis of AH in our study were later diagnosed with carcinoma. In previous studies, up to 50% of women who received biopsy diagnoses of AH had occult endometrial carcinoma when hysterectomy was performed soon thereafter (Silverberg, 2000; Valenzuela *et al*, 2003; Shutter and Wright, 2005; Trimble *et al*, 2006). To minimise including such patients, we excluded cases diagnosed with carcinoma within 1 year of an EH diagnosis, performed a centralised pathology review and excluded cases and controls whose index biopsies were upgraded from EH to carcinoma, and demonstrated robust results in sensitivity analyses that successively excluded risk sets of cases diagnosed within 2, 3, or 4 years of their index biopsies. Our average clinical progression interval was 6 years. Biopsy sampling error and variable timing of repeat biopsies make it difficult to establish exact progression times or distinguish true progression from persistence of undetected, early-stage occult carcinoma (Silverberg, 2000). Nonetheless, women with long intervals between diagnosis of AH and carcinoma probably represent true examples of progression.

Of the 25 potential cases whose index biopsies were originally diagnosed as AH, our pathology panel reclassified 28% as carcinoma, confirmed 24% as AH, and downgraded the severity of 44% to SH, CH, DPEM, or negative. These results resemble findings from a recent Gynecologic Oncology Group (GOG) study of 289 patients who underwent hysterectomy within 12 weeks of receiving a community-based biopsy diagnosis of AH (Trimble *et al*, 2006). The GOG pathology panel upgraded 29% of AH diagnoses to carcinoma, confirmed 40% as AH, and downgraded 26% to less-severe lesions. Hysterectomy revealed invasive carcinoma in 19% of the GOG patients whose original diagnoses were downgraded. In our study, 29% of potential cases – all of whom were later diagnosed with carcinoma – had index biopsies that the panel downgraded to negative. Although AH may be overdiagnosed in community settings (Silverberg, 2000), sampling errors and interpretive challenges commonly beset endometrial biopsy diagnoses.

All patients in our study had at least one endometrial biopsy that was originally classified as EH or DPEM. Most of them received repeat biopsies and progestogen therapy. Approximately 75% of the endometrial carcinomas diagnosed at KPNW between 1970 and 2003 occurred among women who either had no earlier EH or underwent hysterectomy within weeks or months after an EH diagnosis. The well-differentiated stage 1 endometrioid carcinomas (Bokhman, 1983) that formed the majority of our case group are typical of most endometrial carcinomas diagnosed in Europe and North America (Amant *et al*, 2005). Our case patients presumably had a similar clinical course as women whose carcinomas are not preceded by a diagnosis of EH. Our results can be generalised to women who have received biopsy-based diagnoses of EH, who do not undergo hysterectomy for at least 1 year, and who receive management and follow-up in accord with community standards.

High BMI, a history of menstrual irregularities, and less exogenous hormone use were more common in cases than in controls. Statistical adjustment for treatment and minor differences in the repeat biopsies did not substantially affect progression risks, but it remains possible that residual differences contributed to our observations. Varied approaches to clinical follow-up of EH contribute to the inherent difficulties of assessing progression risk (Zaino, 2000). Our results caution that, even with contemporary treatment and surveillance (Montgomery *et al*, 2004; Karamursel *et al*, 2005; McCluggage, 2006), increased risk may persist for many years after EH is diagnosed.

Our analysis could theoretically underestimate true progression risk for non-atypical EH if the EH lesions that were treated with hysterectomy within 1 year (whom we excluded) were markedly more aggressive than those that were included in our analysis, but this seems unlikely. The nearly identical progression intervals across categories of original EH diagnoses suggest that those classifications did not predict subsequent clinical behaviour of non-atypical EH. There were no substantial differences in clinical or patient characteristics at the time of index biopsy that could have been used to reliably identify high-risk SH or CH patients. Cases and controls came from one health plan and likely received similar clinical care after their EH diagnoses. Therefore, the experience of women diagnosed with SH or CH in our study is likely representative of the general population of women with non-atypical EH.

Our study design represents an improvement over previous studies that lacked control groups (Kurman *et al*, 1985; Lindahl and Willen, 1994; Terakawa *et al*, 1997; Tabata *et al*, 2001; Horn *et al*, 2004; Baak *et al*, 2005), included few women with EH who developed carcinoma (Kurman *et al*, 1985; Feldman *et al*, 1994; Lindahl and Willen, 1994; Horn *et al*, 2004; Baak *et al*, 2005) or relied on short follow-up (Terakawa *et al*, 1997; Tabata *et al*, 2001). Previous studies expressed risk as crude percentages – e.g., 20% of patients with non-atypical AH (Ferenczy and Gelfand, 1989) or 29% of patients with AH (Kurman *et al*, 1985) progress to cancer – rather than population-based rate ratios. In our study, 2, 9, and 14% of all women with original community diagnoses of SH, CH, and AH, respectively, were subsequently diagnosed with endometrial carcinoma. Using the pathology panel diagnoses, the cumulative probabilities of progression for SH, CH, and AH were 10, 10, and 40%, respectively. Therefore, previous estimates of carcinoma risk based on consensus diagnoses of SH, CH, and AH (Kurman *et al*, 1985; Ferenczy and Gelfand, 1989; Montgomery *et al*, 2004; Horn *et al*, 2007) may require revision. Our findings reaffirm the need to improve the sensitivity and specificity of AH diagnoses (Soslow, 2006) and efficiently identify the rare non-atypical EH lesions that are likely to progress. Modern outpatient biopsy techniques achieve over 90% sensitivity for detecting carcinoma (Dijkhuizen *et al*, 2000), but better endometrial assessment (Zaino, 2000), histopathologic classifications (Mutter, 2002), and candidate molecular markers (Mutter, 2000) deserve further study.

Our study used one large, essentially population-based health plan with linked data covering 34 years. We reviewed all available pathology from patients with a full range of EH and DPEM and accounted for clinical follow-up, repeat biopsies, and specific treatments. However, few women with panel-confirmed AH had extended follow-up. Our analysis captured clinical progression in the context of contemporary clinical management (Ferenczy and Gelfand, 1989; Randall and Kurman, 1997; Marsden and Hacker, 2001; Montgomery *et al*, 2004; Clark *et al*, 2006; McCluggage, 2006), rather than natural history. Repeated sampling, treatment, and censoring may have affected the absolute risk of carcinoma in our study population compared with untreated women, but our rate ratios are likely unbiased.

In conclusion, the risk that a woman who receives a biopsy diagnosis of AH will progress to carcinoma is high during early and long-term follow-up. The overall progression risks for SH and CH are lower than previously reported, but a small percentage of these patients progress to carcinoma despite conventional clinical follow-up. On the basis of these findings, a dichotomous classification of non-atypical EH *vs* atypical EH, along with refined detection and classification of endometrial carcinoma precursors, could improve clinical management of abnormal vaginal bleeding.

## Figures and Tables

**Table 1:** Follow-up characteristics of eligible cases and eligible controls

	**Cases (*N*=138)**	**Controls (*N*=241)^a^**	***P*-value**
*Age at EH diagnosis (years)*			
<44	28 (20%)	53 (22%)	0.99
45–48	28 (20%)	54 (22%)	
49–52	26 (19%)	40 (17%)	
53–58	29 (21%)	51 (21%)	
59 or older	27 (20%)	43 (18%)	
Mean (years)	52.1	51.5	
Median year at EH diagnosis (range)	1989 (1971–2001)	1989 (1972–2002)	
			
*Age at carcinoma diagnosis or censoring (years)*			0.82
<50	20 (15%)	46 (19%)	
50–54	33 (24%)	52 (22%)	
55–59	25 (18%)	42 (17%)	
60–67	29 (21%)	54 (22%)	
68 or older	31 (22%)	47 (20%)	
			
*Median follow-up interval (years)*	6.7 (1.0–24.5)	6.4 (1.0–24.5)	0.62
By final diagnosis of EH			
DPEM	10.4 (1.3–24.5)	5.9 (1.0–23.4)	
SH	8.6 (1.0–22.1)	6.7 (1.0–24.5)	
CH	5.9 (1.6–18.3)	6.7 (1.2–24.5)	
AH	4.4 (1.0–18.3)	6.7 (1.0–23.4)	
			
*Follow-up biopsies*			
At least one	104 (75%)	207 (86%)	0.02
At least one within 1st 6 months	31 (22%)	128 (53%)	0.01
Median number (range)	2 (0–12)	2 (0–13)	
Mean among women with at least 1	2.9	2.5	0.41
			
*Treatment during progression interval*			
Any progestogen	118 (86%)	222 (92%)	0.17
Oral^b^	99 (72%)	208 (86%)	0.13
Intramuscular^b^	29 (21%)	42 (17%)	0.75

AH=atypical hyperplasia; CH=complex hyperplasia; DPEM=disordered proliferative endometrium; EH=endometrial hyperplasia; SH=simple hyperplasia.

*χ*^2^ Likelihood ratio *P*-values from conditional logistic regression analysis, adjusted for age at index biopsy, date of index biopsy, duration of follow-up, and weighted based on the batch-quota and counter-matched sampling. For progression interval and follow-up biopsies, *P*-value is from *t*-tests.

a Four hundred and thirteen controls were originally matched to cases on age at EH diagnosis, date of EH diagnosis, and duration of follow-up, based on the original community diagnoses of EH. Because the 172 controls who were ineligible based on the pathology panel diagnoses are not included above, the distribution of age, date, and follow-up interval among the 241 eligible controls above differs slightly from the distribution among all 413 potential controls.

b Not mutually exclusive.

**Table 2:** Selected descriptive and clinical factors

	**Cases (*N*=138)**		**Controls (*N*=241)**		
	***N***	**%**	***N***	**%**	***P*-value**
*Age at menarche (years)*					0.35
<12	21	15.2	42	17.4	
12–13	61	44.2	107	44.4	
14 or older	28	20.3	40	16.6	
					
*Ever sought treatment for irregular menses*					0.007
Yes	70	50.7	111	46.1	
No	2	1.5	24	10.0	
					
*Number of pregnancies*					0.02
0	21	15.2	31	12.9	
1	15	10.9	22	9.1	
2–3	56	40.6	109	45.2	
4+	46	33.3	79	32.8	
					
*Number of live births*					0.51
0–1	30	21.7	37	15.4	
2–3	64	46.4	122	50.6	
4+	44	31.9	82	34.0	
					
*BMI at index biopsy (kg m* ^*−2*^ *)*					0.005
<25	26	18.8	70	29.1	
25–29	32	23.2	45	18.7	
30–34	23	16.7	41	17.0	
35–39	26	18.8	24	10.0	
40+	29	21.0	36	14.9	
					
*Menopausal status at index biopsy*					0.59
Pre- or perimenopausal	82	59.4	151	62.7	
Postmenopausal	54	39.1	82	34.0	
Age at menopause ⩽45 years	6	4.4	12	5.0	
Age at menopause 46–52 years	28	20.3	40	16.6	
Age at menopause 53+ years	16	11.6	24	10.0	0.62^a^
					
*Ever diagnosed with diabetes*					0.03
Yes	34	24.6	40	16.6	
No	81	58.7	168	69.7	
					
*Personal history of cancer*					0.70
Yes	16	11.6	19	7.9	
No	113	81.9	196	81.3	
					
*Smoking status at index biopsy*					0.88
Never	77	55.8	133	55.2	
Former	36	26.1	55	22.8	
Current	19	13.8	32	13.3	
					
*Oral contraceptive use before index biopsy*					0.08
Ever	28	20.3	84	34.9	
Never	98	71.0	131	54.4	
					
*Menopausal hormone therapy use*					<0.0001
At index biopsy					
Never	89	64.5	71	29.5	
Former	15	10.9	54	22.4	
Current	25	18.1	75	31.1	
					
At diagnosis/censoring					<0.0001
Never	53	38.4	4	1.7	
Former	50	36.2	102	42.3	
Current	31	22.5	122	50.6	

BMI=body mass index.

Missing values are not shown. *P*-values are *χ*^2^ likelihood ratio *P*-values from conditional logistic regressions, adjusted for age at index biopsy, date of index biopsy and duration of follow-up, and weighted based on the batch-quota and counter-matched sampling.

a *χ*^2^ *P*-value for age at menopause.

**Table 3:** Relative risk of being diagnosed with endometrial carcinoma 1 or more years after a diagnosis of EH

	**Cases**		**Controls**					
**Pathology panel diagnosis for index biopsy**	***N***	**%**	***N***	**%**	**Rate ratio^a^**	**95% CI**	**Rate ratio^b^**	**95% CI**
DPEM	33	23.9	97	40.3	1.0	Ref.	1.0	Ref.
Any EH	105	76.1	144	59.7	3.4	1.9–6.0	4.0	2.0–7.7
								
Non-atypical EH	62	44.9	110	45.6	2.0	1.1–3.8	2.2	1.1–4.7
SH	41	29.7	67	27.8	1.9	0.96–3.9	2.0	0.92–4.5
CH	21	15.2	43	17.8	2.2	0.91–5.3	2.8	1.0–7.9
AH	43	31.2	34	14.1	9.9	4.3–23.1	14.2	5.3–38.0

AH=atypical hyperplasia; CH=complex hyperplasia; CI=confidence interval; DPEM=disordered proliferative endometrium; EH=endometrial hyperplasia; SH=simple hyperplasia.

DPEM is the reference group for all rate ratios. SH includes one case and two controls diagnosed with ‘EH, not otherwise specified’.

a Rate ratios are based on conditional logistic regression analysis adjusted for age at index biopsy, date of index biopsy, interval between EH and carcinoma, and weighted based on the batch-quota and counter-matched sampling. All controls were diagnosed with EH at the same age and date as the cases and remained at risk (i.e., no hysterectomy or uterine cancer) for at least as long as the progression interval of the cases.

b Rate ratios are also adjusted for body mass index (BMI) at the time of EH diagnosis (<25, 25–34, ⩾35 kg m^−2^), progestogen-based treatment for EH (any progestogen-based treatment, other treatment, or no treatment), and follow-up biopsies (any biopsy taken within 6 months of the index biopsy, any subsequent follow-up biopsy, or none).

**Table 4:** Relative risk of being diagnosed with endometrial carcinoma 1 or more years after a diagnosis of EH, stratified by time interval between EH and carcinoma

	**Follow-up interval^a^**							
	**1–4.9 years^b^**				**5 or more years^c^**			
**Pathology panel diagnosis for index biopsy**	**Cases**	**Controls**	**RR^d^**	**95% CI**	**Cases**	**Controls**	**RR^d^**	**95% CI**
DPEM	12	44	1.0	Ref.	21	53	1.0	Ref.
SH	12	31			29	36		
CH	9	13	3.2	0.5–22.2	12	30	1.1	0.4–3.2
AH	24	14	48.0	7.8–294.2	19	20	3.5	1.3–9.6

AH=atypical hyperplasia; CH=complex hyperplasia; CI=confidence interval; DPEM=disordered proliferative endometrium; EH=endometrial hyperplasia; RR=rate ratio; SH=simple hyperplasia.

DPEM or SH is the reference group for all RRs. SH includes one case and two controls diagnosed with ‘EH, not otherwise specified’.

All controls were diagnosed with EH at the same age and date as the cases and remained at risk (i.e., no hysterectomy or diagnosis of uterine cancer) for at least as long as the progression interval of the cases.

a Interval between index biopsy and diagnosis of carcinoma (cases) or matched censoring date (controls). The minimum follow-up interval for all cases and controls was 1 year.

b Restricted to the 57 cases who were diagnosed with carcinoma between 1 and 4.9 years after their diagnosis of EH, plus the 102 controls who were individually matched to these cases on age at EH diagnosis, date of EH diagnosis, and duration of follow-up. See the Materials and Methods section for additional details.

c Restricted to the 81 cases who were diagnosed with carcinoma 5 or more years after their diagnosis of EH, plus the 139 controls who were individually matched to these cases on age at EH diagnosis, date of EH diagnosis, and duration of follow-up. See the Materials and Methods section for additional details.

d Rate ratios are based on conditional logistic regression analysis adjusted for age at the time of index biopsy, date of index biopsy, interval between EH and carcinoma, body mass index (BMI) at the time of EH diagnosis (<25, 25–34, ⩾35 kg m^−2^), progestogen-based treatment for EH (any progestogen-based treatment, other treatment, or no treatment), and follow-up biopsies (any biopsy taken within 6 months of the index biopsy, any subsequent follow-up biopsy, or none), and weighted based on the batch-quota and counter-matched sampling.
