# Supplementary material for: Endometrial carcinoma risk among women diagnosed with endometrial hyperplasia: the 34-year experience in a large health plan
Source: Br J Cancer. 2007 Nov 20;98(1):45–53. doi: 10.1038/sj.bjc.6604102 (PMC2359718; doi:10.1038/sj.bjc.6604102)
Supplement: Supplementary Table 1 [file 6604102x1.doc]

Supplemental Table 1. Original and pathology panel diagnoses for index biopsies from cases

and controls.

|  | Pathology Panel Diagnosis | | | | | |  |  |
| --- | --- | --- | --- | --- | --- | --- | --- | --- |
|  | Negative | DPEM | SH* | CH | AH** | CA |  |  |
| Original Diagnosis |  |  |  |  |  |  |  | Total |
| Potential cases |  |  |  |  |  |  |  |  |
| DPEM | 35 | 6 | 8 | 2 | 5 | 0 |  | 56 |
| SH | 20 | 8 | 11 | 4 | 16 | 2 |  | 64 |
| CH | 19 | 8 | 14 | 9 | 7 | 4 |  | 69 |
| CAH | 2 | 3 | 5 | 1 | 6 | 7 |  | 25 |
| New index biopsy |  | 8 | 4 | 5 | 8 |  |  |  |
| Total: | 76 | 33 | 42 | 21 | 42 | 13 |  | 214 |
|  |  |  |  |  |  |  |  |  |
| Potential controls |  |  |  |  |  |  |  |  |
| SH | 63 | 33 | 18 | 7 | 3 | 0 |  | 129 |
| CH | 50 | 41 | 29 | 20 | 10 | 0 |  | 153 |
| AH | 46 | 23 | 20 | 16 | 21 | 3 |  | 131 |
| Total: | 159 | 97 | 67 | 43 | 34 | 3 |  | 413 |

Negative includes inactive, atrophic, SEM, PEM, and Polyp. CA, carcinoma.

Potential case group includes 189 women who were diagnosed with cancer and 25 women who were diagnosed with CAH at hysterectomy.

Among potential cases, original community diagnoses of SH includes 8 women diagnosed with “EH, NOS” and CAH includes 3 women diagnosed with “AH”; final diagnoses of SH includes 2 women diagnosed with “EH, NOS” and CAH includes 2 women diagnosed with “AH.”

25 of the 76 potential cases with a pathology panel diagnosis of “Negative” were eligible based on a new index biopsy, the final diagnoses for their original index biopsies were atrophic (N=1), inactive (N=5), proliferative endometrium (N=8), polyp (N=1), secretory endometrium (N=5), unsatisfactory / no review (N=3), and slides unavailable (N=2). The original diagnoses for these 25 biopsies were proliferative endometrium (N=3), DPEM (N=4), SH (N=5), CH (N=6), CAH (N=2), inactive (N=1), and other (N=4).

Among potential controls, original community diagnoses of CAH includes 38 women diagnosed with AH and 9 women diagnosed with SAH; community diagnosis of SH includes 1 woman with “EH, NOS”; final diagnosis of SH includes 2 women diagnosed with “EH, NOS”.

Slides from 13 potential cases (4 SH, 8 CH, and 1 CAH) and 10 potential controls (5 SH, 3 CH, and 2 CAH) were unavailable or unsatisfactory for review.
